# CD_8+ _T lymphocytes in lung tissue from patients with idiopathic pulmonary fibrosis

**DOI:** 10.1186/1465-9921-6-81

**Published:** 2005-07-24

**Authors:** Zoe Daniil, Panagiota Kitsanta, George Kapotsis, Maria Mathioudaki, Androniki Kollintza, Marilena Karatza, Joseph Milic-Emili, Charis Roussos, Spyros A Papiris

**Affiliations:** 1 Department of Critical Care and Pulmonary Services, National and Capodistrian University of Athens, "Evangelismos" Hospital, Athens, Greece; 2 Pathology Department, "Evangelismos" Hospital, Athens, Greece; 3 Hematology Department, "Evangelismos" Hospital, Athens, Greece; 4 Meakins-Cristie Laboratories, McGill University, Montreal, Quebec, Canada

## Abstract

**Background:**

Several studies have implicated a role of inflammation in the pathogenesis of lung damage in idiopathic pulmonary fibrosis (IPF). Parenchymal lung damage leads to defects in mechanics and gas exchange and clinically manifests with exertional dyspnea. Investigations of inflammatory cells in IPF have shown that eosinophils, neutrophils and CD_8+ _TLs may be associated with worse prognosis. We wished to investigate by quantitative immunohistochemistry infiltrating macrophages, neutrophils and T lymphocytes (TLs) subpopulations (CD_3+_, CD_4+ _and CD_8+_) in lung tissue of patients with IPF and their correlation with lung function indices and grade of dyspnoea.

**Methods:**

Surgical biopsies of 12 patients with IPF were immunohistochemically stained with mouse monoclonal antibodies (anti-CD_68 _for macrophages, anti-elastase for neutrophils, and anti-CD_3_, anti-CD_4_, anti-CD_8 _for CD_3+_TLs, CD_4+_TLs, and CD_8+_TLs respectively). The number of positively stained cells was determined by observer-interactive computerized image analysis (SAMBA microscopic image processor). Cell numbers were expressed in percentage of immunopositive nuclear surface in relation to the total nuclear surface of infiltrative cells within the tissue (labeling Index). Correlations were performed between cell numbers and physiological indices [FEV_1_, FVC, TLC, *D*LCO, PaO_2_, PaCO_2 _and P(A-a)O_2_)] as well as dyspnoea scores assessed by the Medical Research Council (MRC) scale.

**Results:**

Elastase positive cells accounted for the 7.04% ± 1.1 of total cells, CD_68+ _cells for the 16.6% ± 2, CD_3+ _TLs for the 28.8% ± 7, CD_4+ _TLs for the 14.5 ± 4 and CD_8+ _TLs for the 13.8 ± 4. CD_8+_TLs correlated inversely with FVC % predicted (r_s _= -0.67, p = 0.01), TLC % predicted (r_s _= -0.68, p = 0.01), DLCO % predicted (r_s _= -0.61, p = 0.04), and PaO_2 _(r_s _= -0.60, p = 0.04). Positive correlations were found between CD_8+_TLs and P(A-a)O_2 _(r_s _= 0.65, p = 0.02) and CD_8+_TLs and MRC score (r_s _= 0.63, p = 0.02). Additionally, CD_68+ _cells presented negative correlations with both FVC % predicted (r_s _= -0.80, p = 0.002) and FEV_1 _% predicted (r_s _= -0.68, p = 0.01).

**Conclusion:**

In UIP/IPF tissue infiltrating mononuclear cells and especially CD_8+ _TLs are associated with the grade of dyspnoea and functional parameters of disease severity implicating that they might play a role in its pathogenesis.

## Background

In usual interstitial pneumonia (UIP)/idiopathic pulmonary fibrosis (IPF) the role of inflammation in the pathogenesis of fibrosis is debatable [1-3]. Traditionally, UIP/IPF was regarded to develop in response to chronic inflammation of the lung parenchyma [4]. This view was advanced from previous studies implicating a role of the inflammatory cells including neutrophils, macrophages, eosinophils and T lymphocytes (TLs), based on the observation of their accumulation in sites of disease activity [5-8] or on their presence in high numbers in bronchoalveolar lavage [9-12].

Actually, the current pathogenetic theory that holds in UIP/IPF, implicates that fibrosis per se might progress despite a paucity of interstitial inflammation [13]. However, even in this case, recent data still indicate the contention that the type of the inflammatory response may modulate tissue injury, fibrosis or both [3,4]. Animal studies imply that TLs might play a role in the initiation and the evolution of pulmonary fibrosis. They also suggest that different TLs subpopulations, including both CD_4+ _and CD_8+ _subsets, might contribute through their ability to secrete fibrogenic cytokines [14,15]. Ultimately, in UIP/IPF, the inflammatory response is considered to resemble closely the type-2 T lymphocytic pattern [16-18] and drives the process in a profibrogenic direction.

The present study was designed to investigate by quantitative immunohistochemistry the inflammatory cell pattern in lung tissue of patients with UIP/IPF (macrophages, neutrophils, and CD_3+_, CD_4+_, CD_8+ _TLs) and to correlate their population numbers with the lung function indices and grade of dyspnoea.

## Methods

### 1. Subjects

The study population consisted of 12 untreated patients with IPF and included 7 ex-smokers, and 5 never smokers (Table 1). They were recruited from the respiratory outpatient clinic of the "Evangelismos" General Hospital, Athens, Greece over a period of 3 years. The diagnosis of UIP/IPF was based on standard criteria [19], which included clinical findings (exertional dyspnoea, non-productive cough, fine bibasilar inspiratory crackles), pulmonary function tests (restrictive pattern and impaired gas exchange), and high resolution computerized tomography findings (bibasilar reticular abnormalities with minimal ground-glass opacities consistent with the diagnosis of IPF). The diagnosis of UIP/IPF was confirmed by video-assisted thoracoscopic lung biopsy in all patients. Pathology examination of these specimens clearly documented UIP according to Katzenstein's and American Thoracic Society – European Respiratory Society criteria (histologic variation with alternating zones of interstitial fibrosis, inflammation, honeycomb change, and normal lung [1,19]. A right thoracic approach was done and two or three samples were taken from the right lower or middle lobe in the region of the greater fissure. Biopsy of the lingular tip was avoided, as changes in this area may be particularly advanced and unrepresentative. All patients experienced a normal and uncomplicated postoperative course. Secondary causes of lung fibrosis were excluded: none of the patients included in this study had a history of environmental or occupational exposure, drug toxicity or connective tissue disease, as documented by patient's history and thorough clinical and immunological work out. The study was approved by the institutional ethics committee and informed consent was obtained from each patient.

**Table 1:** Demographic, clinical and lung function data of all patients at presentation

**Variables**	**Values**
Age (yr)	60 ± 2
Sex (M/F)	5/7
MRC dyspnoea score	1.8 ± 0.3
FEV_1 _(% pr)	85 ± 4
FVC (% pr)	78 ± 4
FEV_1_/FVC (ratio × 100)	85 ± 4
TLC (% pr)	64 ± 3
RV (% pr)	54 ± 4
*D*LCO (% pr)	50 ± 5
PaO_2 _(mmHg)	75 ± 2
P(A-a)O_2_	30 ± 3
PaCO_2 _(mmHg)	36 ± 3

Data are presented as means ± SEM; M = Male; F = Female; pr: predicted

### 2. Pulmonary function tests

The pulmonary function tests included FEV_1_, FVC, FEV_1_/FVC ratio × 100, total lung capacity (TLC), residual volume (RV) and carbon monoxide transfer factor (*D*LCO). TLC and RV were measured by the helium dilution method with a Master Screen apparatus (Erich Jaeger GmbH, Wuerzburg, Germany), and *D*LCO by the single breathholding helium dilution method [20,21]. Lung function measurements (Table 1) were expressed as percentages of predicted values [20,21]. In all patients, the arterial PaO_2 _and PaCO_2 _were also measured at rest, and P(A-a)O_2 _calculated.

### 3. Dyspnea

Dyspnea was assessed with the modified (6-point) MRC dyspnoea self-administered questionnaire [22] that consists of six questions about perceived breathlessness: category 0, no dyspnoea; category 1, slight degree of dyspnoea (troubled by shortness of breath when hurrying on the level or walking up a slight hill); category 2, moderate degree of dyspnoea (walks slower than people of the same age on the level because of breathlessness); category 3, moderately severe degree of dyspnoea (has to stop because of breathlessness when walking at own pace on the level); category 4, severe degree of dyspnoea (stops for breath after walking about 100 yards or after a few minutes on the level); category 5, very severe degree of dyspnoea (too breathless to leave the house or breathless when dressing or undressing).

### 4. Histology

Open lung biopsies from the 12 patients were used. They were taken for diagnostic and staging purposes and were analyzed according to the Katzenstein's criteria [1] by two pathologists. Specimens were fixed in 4% formalin and after dehydration embedded in paraffin. Tissue sections were orientated and serial sections of 4 μm thickness were cut and immunohistochemistry was performed according to the Streptavidin-Biotin method.

### 5. Immunohistochemistry

To evaluate macrophages, neutrophils and the lymphocyte-subpopulations in lung tissues, 4 μm paraffin sections were immunohistochemically stained with the following mouse monoclonal antibodies (all from DAKO, Glostrup, Denmark): macrophages-histiocytes with anti CD_68_, (clone: M814, dilution 1:4000), neutrophils with anti-elastase (clone:M752 dilution 1:4000), T-cells with anti-CD_3 _(dilution 1:200), anti-CD_4 _(dilution 1:100), and anti-CD_8 _(dilution 1:4), according to the labeled Streptavidin-Biotin Complex method. The sections were deparaffinized and rehydrated with Tris-Buffered Saline (TBS: 0.005 M Tris, 0.15 M NaCl), pH = 7.6 for 10 minutes. Endogenous peroxidase was blocked with 3% hydrogen peroxide for 5 minutes. Then they were washed in TBS and incubated with primary antibodies at the appropriate dilutions for one hour. Biotinylated antimouse IgG was used as a secondary antibody (DAKO), followed by peroxidase-conjugated streptavidin (DAKO). The peroxidase reaction was developed using 3,3'-diaminbenzidine tetrachloride (0.25 mg dissolved in 1 ml of 0.02% hydrogen peroxide) for 3 min.

### 6. Lung Parenchyma Computer Image Analysis

The number of positively stained cells was determined by observer-interactive computerized image analysis (SAMBA microscopic image processor), whose hardware and software have been described by Brugal and associates [23]. This system is fitted with a standard Zeiss axioplan microscope, a color video camera (Sony Corporation Tokyo, Japan), an image analysis processor (matrox) and an IBM compatible Pentium 2, 166 MHZ computer. Estimation of the standard error of the mean within 95% confidence limits required a maximum of at least randomly selected 15 High Power fields (X400-Zeiss microscope) (Analysis per area of approximately 110000 μm^2^). The immunostaining was analyzed as dark brown color with counterstained cells as false blue. Formal scoring (labeling Index) for each antibody was then performed in one section for each paraffin block. Interobserver variability was very low (<0.03%). The results were expressed in percentage of immunopositive nuclear surface in relation to the total nuclear surface of infiltrative cells within the tissue (labeling Index), as previously described [24,25]. Blood vessels, connective tissue and cartilage structures were excluded.

### 7. Statistical Analysis

Data were expressed as means and standard error (SEM). Correlation coefficients were calculated using Spearman's rank method. A p-value of less than 0.05 was considered statistically significant. Analysis was performed using the SAS System software.

## Results

Demographic characteristics, MRC dyspnoea score and lung function data of all patients are listed in Table 1. All patients claimed some degree of dyspnoea (MRC score > 0) and most patients had a restrictive lung function pattern characterized by a decrease in TLC (mean value was 64% of predicted) and an increased in FEV_1_/FVC ratio ×100 (mean value was 85% of predicted). The *D*LCO was decreased in all patients (mean value was 50% of predicted).

**Figure 1:**
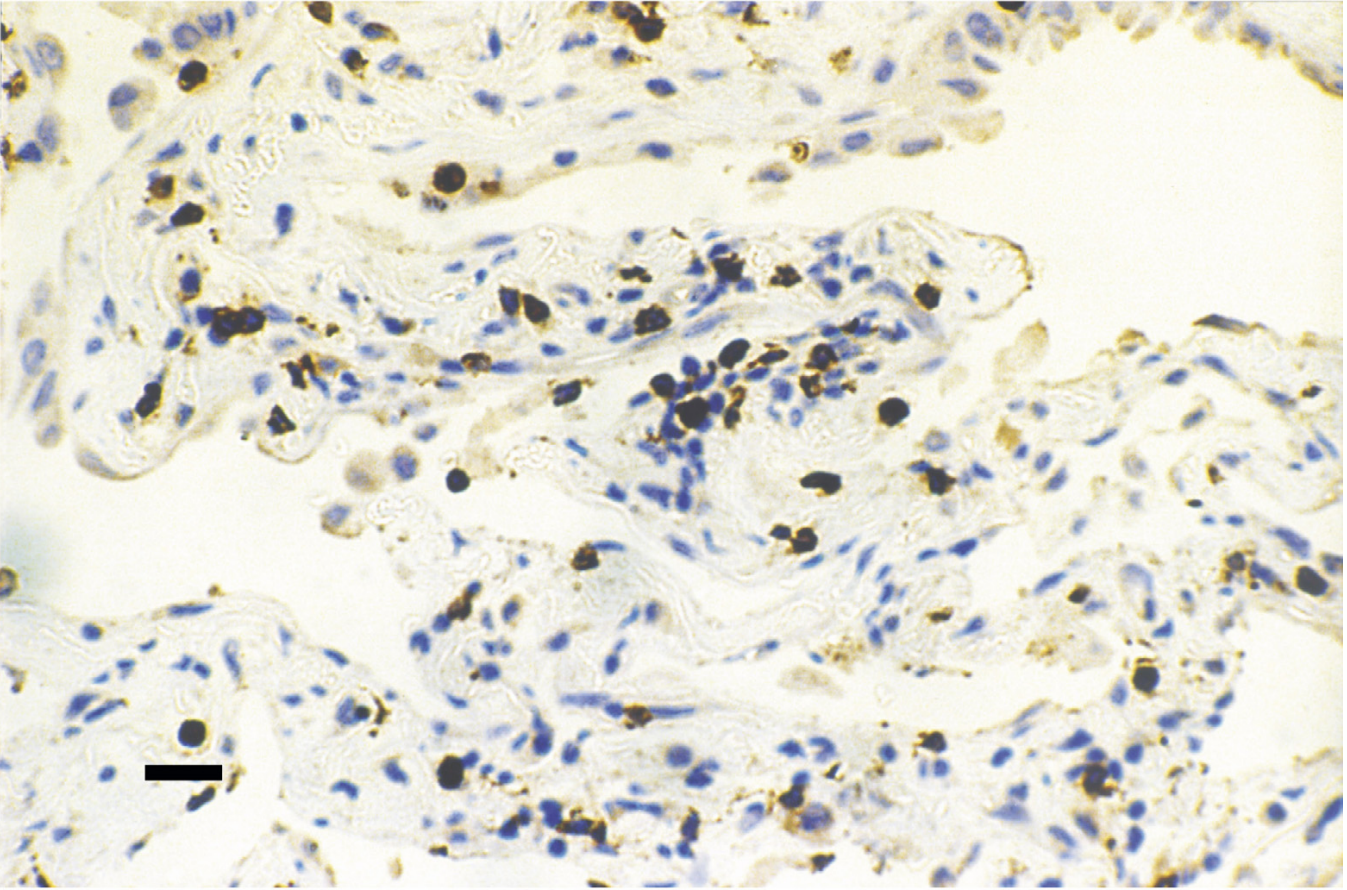
The CD_8+_TLs infiltrate diffusely the lung parenchyma (scale bar = 25 μm).

Among the inflammatory cells studied T lymphocytes (CD_3+_) appeared as the most numerous cells infiltrating the lung parenchyma. They were found in aggregates, within lymphoid follicles or diffusely within the fibrotic lung parenchyma and mainly in the areas with moderate or severe thickening of the alveolar wall. They were also observed within the wall of the alveoli. The CD_4+ _subpopulation was found in aggregates inside or around lymphoid follicles. A little portion of them infiltrated the lung parenchyma diffusely, especially the alveolar wall. On the other hand, the CD_8+ _cells infiltrated the parenchyma mainly diffusely (Fig 1); they were also found within the alveolar wall, around the fibrotic foci and in the areas with alveolar thickening. Less commonly they were distributed within aggregates or lymphoid follicles. Macrophages (CD_68+ _cells) were preferentially located in the lamina propria of the airways compared with the surface epithelium and the submucosa. They were also distributed in large aggregates in the dilated alveolar spaces. Neutrophils (Elastase+) were mainly observed in the surface epithelium and within the alveolar wall.

**Figure 2:**
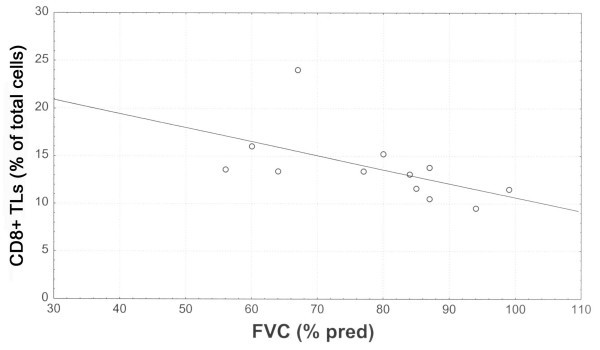
Relationship between CD_8+_TLs (% of total cells) and FVC (% predicted), (r_s _= -0.67, p = 0.01).

**Figure 3:**
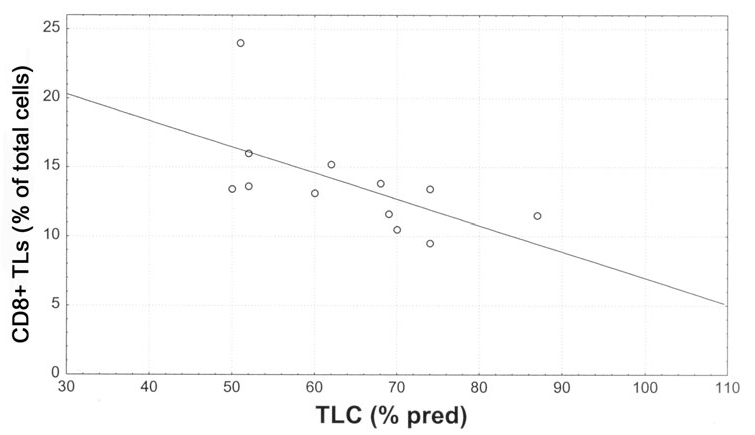
Relationship between CD_8+_TLs (% of total cells) and TLC (% predicted), (r_s _= -0.68, p = 0.01).

**Figure 4:**
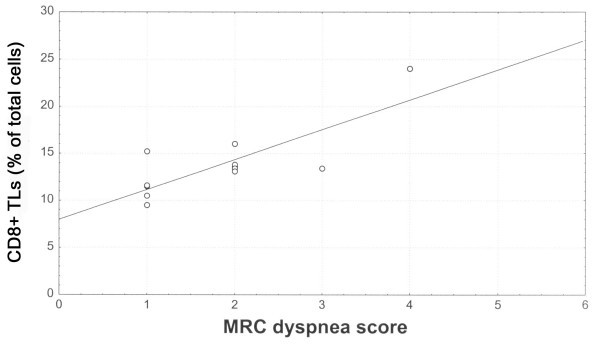
Relationship between CD_8+_TLs (% of total cells) and MRC dyspnea score, (r_s _= 0.63, p = 0.02).

Elastase positive cells accounted for the 7.04% ± 1.1 of total cells, CD_68+ _cells for the 16.6% ± 2, CD_3+ _TLs for the 28.8% ± 7, CD_4+ _TLs for the 14.5 ± 4 and CD_8+ _TLs for the 13.8 ± 4. Among the infiltrating inflammatory cells, CD_8+_TLs were inversely correlated with FVC % predicted (r_s _= -0.67, p = 0.01) (Fig 2), TLC % predicted (r_s _= -0.68, p = 0.01) (Fig 3), *D*LCO % predicted (r_s _= -0.61, p = 0.04), PaO_2 _(r_s _= -0.60, p = 0.04). Positive and statistically significant correlations were found between CD_8+_TLs and P(A-a)O_2 _(r_s _= 0.65, p = 0.02) and CD_8+_TLs and MRC score (r_s _= 0.63, p = 0.02) (Fig 4). Additionally, the CD_68+ _cells presented significant negative correlations with the FVC % predicted (r_s _= -0.80, p = 0.002) and the FEV_1 _% predicted (r_s _= -0.68, p = 0.01). Elastase positive cells, CD_3+ _TLs, and the CD_4+ _TLs did not correlate with any of the functional indices as well as the MRC score.

## Discussion

Several studies have implicated a role of inflammation in the pathogenesis of lung damage in IPF. Parenchymal lung damage leads to defects in mechanics and gas exchange and consequently to exertional dyspnoea, the most prominent and disabling symptom in these patients. This study shows that the type of the inflammatory infiltrate in lung tissue of patients with UIP/IPF was predominantly mononuclear, and that among the different inflammatory cells, CD_8+ _TLs correlated significantly with both functional [FVC, TLC, *D*LCO, PaO_2_, P(A-a)O_2_] and clinical indices (the MRC chronic dyspnoea score) of the disease severity and extent studied and macrophages (CD_68+ _cells) with some of the functional indices studied (FVC and FEV_1_).

Previous studies have shown that a patchy chronic interstitial inflammatory process coexists with an abnormal extracellular matrix deposition, foci of fibroblasts, and alveolar collapse in lung tissue of subjects with IPF [1,2,8,26]. This inflammatory component in tissue specimens appeared to consist primarily of mononuclear cells (macrophages, lymphocytes and plasma cells) while the presence of the other inflammatory cells such as neutrophils and eosinophils appeared less numerous [5,8,26,27]. Previous studies of immunohistochemical analysis including the TLs subpopulations of lung tissue in IPF [5,8,28,29] and other interstitial pneumonias [30] have shown that the inflammatory process is mainly mononuclear, and that both CD_4+ _and CD_8+ _TLs were well represented and diffusely distributed in the interstitium, with an additional component of the CD_4+ _TLs observed inside lymphoid follicles. Our findings do not contrast these observations.

Few studies have attempted to evaluate the structure-function relationship in IPF, and their findings are not always in agreement [31-35]. The selection of patients and the different methodologies might, at least in part, explain discrepancies. Our findings come into agreement with the studies by Fulmer and coworkers [31] and Chinet and coworkers [35] who found significant correlations between the degree of inflammation and lung volumes and some index of gas exchange. Gaensler and Carrington [34] also reported correlations between physiological indices and an estimate of functional impairment from histology (designated by them as pathological severity) in a large but mixed population (502 patients) with "interstitial lung disorders" including 64 patients with UIP. However, comparisons with previous studies are not always possible for the following reasons. First, we used a different methodology than the above-cited works. This is the first study attempted to correlate cell counts including TLs subpopulations in tissue biopsies with clinical and physiological parameters. This should be in relation to the fact that reliable cell counts were not feasible with the past technologies. Second, older studies might have included a mixed population of patients with UIP/IPF, patients with non-specific interstitial pneumonia and patients with pulmonary fibrosis associated with collagen vascular disorders since there were not defined strict criteria for these entities, by that time [36]. Finally, the patients' selection and the effect of previous treatment might have influenced the results.

Inflammatory cells including subpopulations of TLs have been also studied in IPF by bronchoalveolar lavage (BAL) [4] and many of the conclusions regarding the role of inflammation in interstitial lung disorders have been drawn from these studies. The possible role of lymphocytes in the pathogenesis of IPF traditionally received little investigation since increase in their number is an uncommon finding in BAL samples. Hence, early BAL studies have driven attention into neutrophils as well as macrophages. However, evidence from animal models appears to suggest that lymphocytes do play a role in fibrosis [4]. Furthermore, studies on BAL lymphocytes have shown that CD_8+ _TLs are prominent in BAL in IPF [36] and may also be associated with a worse prognosis [37]. T lymphocytes and their phenotypic and functional characteristics have been more extensively studied in scleroderma fibrosis [38-43]. IPF and scleroderma fibrosis are two fibroses with different prognoses [41]. This might be related to the fact that most patients with scleroderma develop a less aggressive form of fibrosing interstitial pneumonia, the non-specific interstitial pneumonia (NSIP) [42]. Indeed, studies that compared the prognosis of patients with "idiopathic" NSIP to that of patients with usual interstitial pneumonia type/IPF have clearly shown that the former present a far better prognosis than the latter [43]. Recent studies with BAL in scleroderma patients have shown that a subset of them, who present more than 15% lymphocytes in BAL [38], or have activated, long-lived CD_8+ _T cells [40], or produce type 2 cytokines (IL-4 and IL-5) by the CD_8+ _TLs [39], present a more aggressive form of interstitial pneumonia. Hence, TLs and in particular the CD_8+ _subset may be associated with progressive fibrosis in scleroderma resembling more patients with IPF.

Notwithstanding correlations do not imply direct cause-effect relationships, we think that the significant negative correlations observed in this group of patients with IPF between CD_8+_TLs and functional indices and the positive correlation observed between the same cells and clinical indices estimating the disease severity as well as the correlation between CD_68+ _cells with FVC and FEV_1 _might suggest a potential pathophysiologic relevance for mononuclear cells and especially CD_8+_TLs in the pathogenesis of pulmonary fibrosis. However, the mechanisms related to these correlations and the relationship of inflammatory and immune parameters to structural changes in the lung parenchyma still remain unknown and further studies are needed for their clarification.

The increase in CD_8+ _TLs observed in lung surgical biopsies in patients with IPF appears intriguing. Classically, the major role of CD_8+ _TLs in the inflammatory response has been considered the rapid resolution of viral infections [44]. It has also become evident that CD_8+ _TLs may contribute to lung injury [45,46]. Viruses have been implicated in the pathogenesis of IPF, and a higher incidence of viral infections (Ebstein Barr Virus, influenza, cytomegalovirus, and possibly Hepatitis C virus) has been reported in these patients [19]. Recently, it has been hypothesized that in patients with IPF an excessive recruitment of CD_8+ _TLs may occur in response to recurrent or persistent viral infections, and this excessive response may play a role for the development of lung damage [47]. The above hypothesis has received some experimental confirmation by the studies of Enelow and coworkers [47] and Small and coworkers [48] who have shown that antigen-specific CD_8+ _T cell recognition of an alveolar epithelial "autoantigen" is sufficient to trigger an inflammatory cascade that results in the histological and physiological manifestations of interstitial pneumonia. CD_8+ _TLs can differentiate into cells that make IFN-γ but no IL-4 (Tc1 cells) promoting attenuation of fibrosis and cells that make IL-4 but not IFN-γ (Tc2 cells) leading to exuberant fibrosis [49]. Though further studies are necessary to address the specific role of the Tc2 cells in pulmonary fibrosis, some data such as the upregulation of genes encoding immunoglobulins and extracellular matrix proteins in IPF lung tissue [50] appear to suggest that the predominance of type-2 immune response in IPF [51] is what drives the process in profibrogenic direction.

## Conclusion

We found that the type of the inflammatory cell infiltrate in surgical biopsies of patients with UIP/IPF was predominantly mononuclear. Among the different inflammatory cells revealed by immunohistochemistry, the CD_8+_TLs correlated significantly with both functional [FVC, TLC, *D*LCO, PaO_2_, P(A-a)O_2_] and clinical indices (the MRC chronic dyspnoea score) of disease severity and extent studied and the CD_68+ _cells with FVC and FEV_1_. These data might suggest a potential role for mononuclear cells and especially CD_8+_TLs in the pathogenesis of pulmonary fibrosis. However, because of the relatively small size of the population studied, further studies are needed to support our findings.

## Competing interests

The author(s) declare that they have no competing interests.

## Authors' contributions

ZD participated in the design of the study and collection of the clinical data, performed the statistical analysis and drafted the manuscript. PK carried out the histology and the immunohistochemical analysis and revised the article. GK participated in the collection of the data and helped to draft the manuscript. MD helped in biopsy evaluation, diagnosis and analysis. AK participated in tissue collection and data analysis. MK participated in data analysis. JM-E helped in the interpretatiuon of the data and revised the article. CR participated in the interpretation of the data and revised the article. SP conceived of the study, participated in its design and coordination and helped to draft the manuscript. All authors read and approved the final manuscript.
